# Identification of a New Morpholine Scaffold as a P2Y12 Receptor Antagonist

**DOI:** 10.3390/molecules21091114

**Published:** 2016-08-24

**Authors:** Young Ha Ahn, Joo-Youn Lee, Hee Dong Park, Tae Hun Kim, Min Chul Park, Gildon Choi, Sunghoon Kim

**Affiliations:** 1 College of Pharmacy, Seoul National University, Seoul 08826, Korea; larica@snu.ac.kr (Y.H.A.); leejy@krict.re.kr (J.-Y.L.); 2 Korea Chemical Bank, Korea Research Institute of Chemical Technology, Daejeon 34114, Korea; 3 LG Life Sciences Ltd., R & D Park, Daejeon 305-343, Korea; hdparkc@lgls.com (H.D.P.); biokth@lgls.com (T.H.K.); 4 Medicinal Bioconvergence Research Center, Department of Molecular Medicine and Biopharmaceutical Sciences, Graduate School of Convergence Science and Technology, Seoul National University, Suwon 16229, Korea; parkmin2@snu.ac.kr; 5 Advanced Institutes of Convergence Technology, Seoul National University, Suwon 16229, Korea; 6 Center for Drug Discovery Technology, Korea Research Institute of Chemical Technology, Daejeon 34114, Korea; gchoi@krict.re.kr

**Keywords:** P2Y12 receptor antagonist, antiplatelet, morpholine moiety, ticagrelor

## Abstract

The P2Y12 receptor is critical for platelet activation and is an attractive drug target for the prevention of atherothrombotic events. Despite the proven antithrombotic efficacy of P2Y12 inhibitors, these thienopyridine scaffolds are prodrugs that lack important features of the ideal antithrombotic agent. For this reason, ticagrelor—a new chemical class of P2Y12 receptor antagonist—was developed, but it can cause shortness of breath and various types of bleeding. Moreover, ticagrelor is a cytochrome P450 3A4 substrate/inhibitor and, therefore, caution should be exercised when it is used concomitantly with strong CYP3A4 inducers/inhibitors. There is a need for novel P2Y12 receptor antagonist scaffolds that are reversible and have high efficacy without associated side effects. Here, we describe a novel antagonist containing a morpholine moiety that was identified by screening libraries of commercially available compounds. The molecule, Compound **E**, acted on P2Y12, but not P2Y1 and P2Y13, and exhibited pharmacological characteristics that were distinct from those of ticagrelor, acting instead on P2Y12 via an allosteric mechanism. These results provide a basis for the development/optimization of a new class of P2Y12 antagonists.

## 1. Introduction

Platelets are essential for hemostasis, which protects the body by stopping bleeding from damaged blood vessels. Abnormal hemostasis can lead to the formation of blood clots due to platelet aggregation, for instance after hematogenous reconstruction. There is, therefore, a need to develop a platelet inhibitor that is both efficacious and stable. Antiplatelet drugs, such as ticagrelor and thienopyridine derivatives (prasugrel, ticlopidine, and clopidogrel), are used in myocardial infarction (MI) and stroke in patients with acute coronary syndrome (ACS) or a history of myocardial infarction [1,2].

Adenosine 5′-diphosphate (ADP) is an important mediator of platelet activation and aggregation [3]. The ADP signal is transduced via interaction with Gq-coupled P2Y1 receptor and Gi-coupled P2Y12 receptor. These receptors are involved in Ca^2+^-dependent cell migration, regulation of cell morphology, and cellular aggregation through adenylyl cyclase inhibition in response to the ADP signal. P2Y1 and P2Y12 must be activated for ADP—induced platelet aggregation, which can be blocked by an agent that independently inhibits these receptors. Unlike P2Y1, P2Y12 has a specific tissue distribution, making it an attractive target for therapeutic intervention. Indeed, P2Y12 is targeted by antithrombotic agents [4], and a variety of P2Y12 receptor antagonists have been reported to inhibit platelet aggregation and exhibit antithrombotic effects, including thienopyridine, ticlopidine, clopidogrel, and CS-747.

Despite their proven antithrombotic effects, thienopyridines scaffolds are prodrugs that lack important features [5,6]. Ticagrelor belongs to a new chemical class known as cyclopentyl-triazolo-pyrimidines that is chemically distinct from thienopyridines. The Platelet Inhibition and Patient Outcomes (PLATO) trials showed that ticagrelor had antithrombotic effects that were superior to those of clopidogrel in patients with acute coronary syndromes [7]. However, side effects of ticagrelor include shortness of breath (dyspnea; 14%) and various types of bleeding, ventricular pauses, hyperuricemia, and elevation of serum creatinine have been reported during ticagrelor treatment [8]. Ticagrelor is a CYP3A4 substrate/inhibitor that participates in drug-drug interactions; indeed, concomitant use with strong CYP3A4 inhibitors, such as ketoconazole, itraconazole, clarithromycin, ritonavir, and telithromycin, is contraindicated, while its co-administration with potent CYP3A inducers, such as carbamazepine, rifampicin, phenytoin, and phenobarbital is discouraged [9]. As such, there is a need for novel P2Y12 receptor antagonist scaffolds that are reversible and have high efficacy without associated side effects.

In this study, we describe a novel P2Y12 receptor antagonist scaffold containing a morpholine moiety that was identified by screening libraries of commercially available compounds. Our findings indicate that this compound can be an effective alternative drug for the prevention of atherothrombotic events.

## 2. Results

### 2.1. High-Throughput Screening of Anti-Platelet Compounds

**Figure 1:**
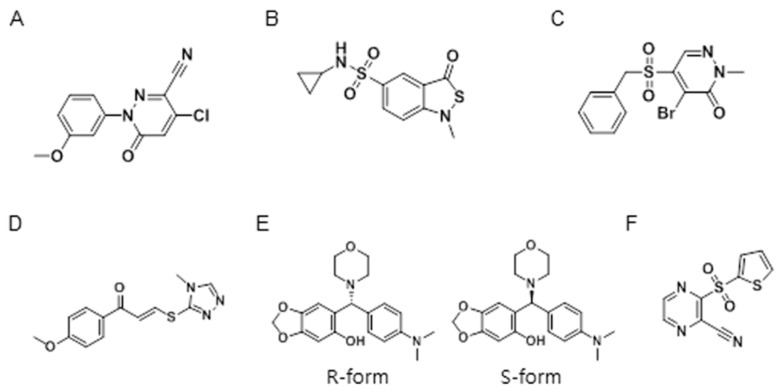
High-throughput screen of anti-platelet compounds. Commercially available compound libraries were screened to identify antagonists of the ADP receptor P2Y12. Six compounds were found to inhibit 20 μM ADP-induced platelet aggregation in a 96-well format. Compounds **A** and **C** contain a pyridazinone moiety. Compounds **B**, **D**, **E**, and **F** contain sulfonamide, triazoline, morpholine, and pyrazine moieties, respectively.

A total of 6211 compounds were offered from LG Life Sciences Ltd. (Daejeon, Chungnam, Korea). All library compounds were dissolved in dimethyl sulfoxide (DMSO), which did not affect platelet aggregation at concentrations below 7% (data not shown). Test compounds (30 μM in 1% DMSO) and platelets were added to the 96-well plate using a multi-drop reagent dispenser, followed by 20 μM ADP; changes in the optical density of platelets were then measured. A total of 43 compounds were found to inhibit ADP-induced platelet aggregation in the initial screen using a cut-off value of 70% inhibition (0.69% hit rate). Six representative compounds were selected and confirmed to inhibit ADP-induced platelet aggregation; their structures are shown in Figure 1.

### 2.2. Anti-Platelet Activities of Selected Compounds

**Figure 2:**
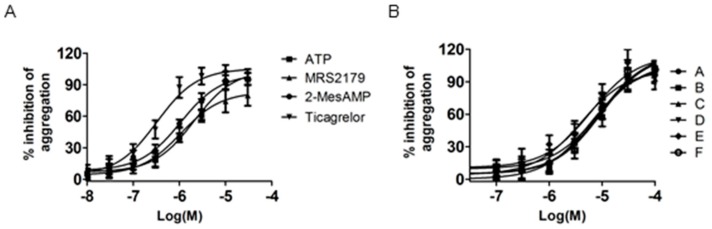
Inhibition of ADP-induced platelet aggregation by six candidate antagonists of P2Y12. The reference compounds ATP, MRS2179, 2-MeSAMP, and AZD6140 were used as ADP, P2Y1, and P2Y12 receptor antagonists. (**A**) references and (**B**) the six candidate compounds inhibited platelet aggregation induced by 20 μM ADP. Data are representative of two experiments performed in triplicate and are expressed as mean ± SD (see also Table 1).

The human platelet aggregation test was used to determine whether a test compound was an ADP receptor agonist or an antagonist. Traditional light transmission aggregometry is considered as the gold standard assay, while the 96-well plate format founded on similar principles provides a high-throughput screening method [10,11]. ADP concentration-dependent platelet stimulation was determined in a 96-well plate; the EC50 of ADP was 4.2 μM. To validate these findings, P2Y1 (MRS2179), P2Y12 (2-MeSAMP and AZD6140), and ADP receptor antagonist (ATP) were used as reference compounds. All of these, as well as the six compounds identified in the screen, inhibited ADP-induced platelet aggregation in a dose-dependent manner (Figure 2A,B). The IC50 values are listed in Table 1.

### 2.3. Binding Affinities of Identified Compounds for the Rhp2y12 Receptor

**Figure 3:**
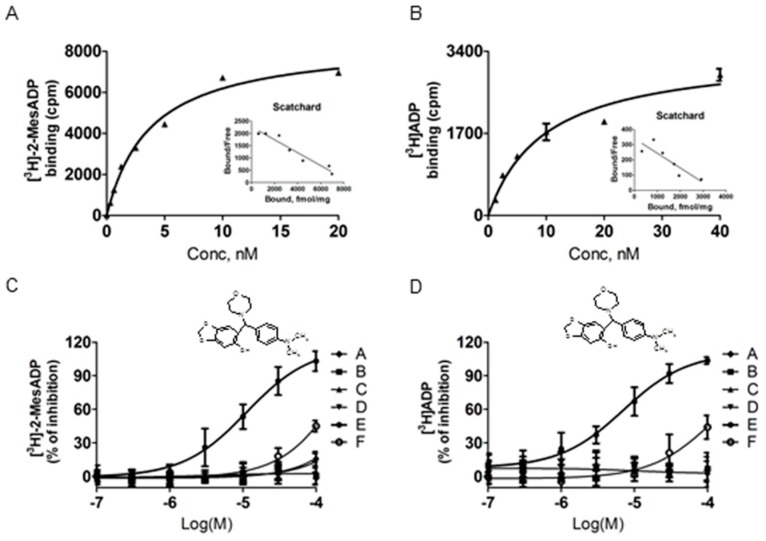
Binding characteristics of rhP2Y12. Saturation curves and Scatchard plots of (**A**) [^3^H]-2-MeSADP and (**B**) [^3^H]ADP. Non-specific binding was determined using 25 µM 2-MeSADP. Membranes prepared from HEK293T cells stably expressing human P2Y12 receptor were incubated with (**C**) 1 nM [^3^H]-2-MeSADP and (**D**) 3 nM [^3^H]ADP with increasing concentrations of test compound to determine IC_50_. Compound **E** (morpholine moiety) selectively inhibited the binding of both radio-ligands. Data are representative of three experiments performed in duplicate or triplicate and are expressed as mean ± SD (see also Table 2).

Six compounds were tested for their ability to inhibit the binding of [^3^H]-2-MeSADP and [^3^H]ADP to rhP2Y12. The specific binding was saturable and the Scatchard plot analysis yielded a linear fit. The binding sites (B_max_) and K_d_ values of rhP2Y12 were 8502 ± 247 fmol/mg and 3.74 nM for [^3^H]-2-MesADP (Figure 3A) and 3378 ± 241 fmol/mg and 9.7 nM for [^3^H]ADP (Figure 3B). Among the six compounds, Compound **E** (6-[(4-dimethylamino-phenyl)-morpholin-4-yl-methyl]-benzo[1,3]dioxol-5-ol) selectively inhibited the binding of both [^3^H]-2-MesADP and [^3^H]ADP, with IC_50_ values of 15.5 and 8.3 μM, respectively. Compound **F** (3-[Thiphene-2-sulfonyl]-pyrazine-2-carbonitrile), but none of the other compounds, slightly inhibited the binding of both ligands to rhP2Y12 (Figure 3C,D).

### 2.4. Comparison of the Pharmacological Characteristics of Compound **E** and Ticagrelor

**Figure 4:**
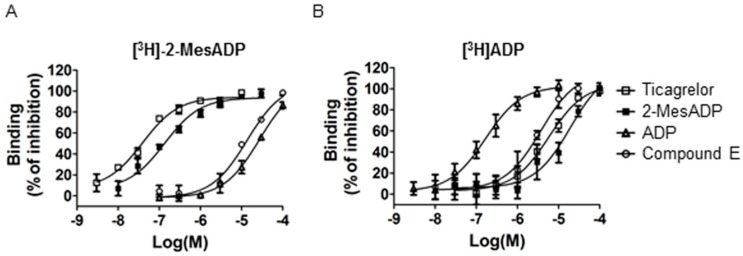
Comparison of pharmacological characteristics of Compound E and ticagrelor. Competitive binding was determined using (**A**) [^3^H]-2-MeSADP and (**B**) [^3^H]ADP as radio-ligands. Membranes prepared from HEK293T-rhP2Y12 cells were incubated with ticagrelor, 2-MeSADP, ADP, and Compound **E** with increasing concentrations of test compound to determine IC_50_. Data are representative of three experiments performed in duplicate or triplicate and are expressed as mean ± SD (see also Table 3).

Ticagrelor is the first identified reversibly binding oral P2Y12 antagonist; it exhibits non-competitive antagonism of [3] ADP, but competes for receptor binding with [3H]2-MeSADP [12,13]. To characterize the affinity of Compound **E** for P2Y12, we compared the binding affinities of 2-MeSADP, ADP, ticagrelor, and Compound **E** using [^3^H]ADP and [^3^H]-2-MeSADP (Figure 4 and Table 2). ADP and 2-MeSADP competed with [3H]ADP, with the former showing a 90-fold higher potency. Ticagrelor also displaced [3H]ADP with an IC_50_ of 8.4 μM, which was 210-fold lower than for [3H]2-MeSADP (IC_50_ = 0.04 μM). Compound **E** displaced both [3H]ADP and [3H]2-MeSADP, with a 1.9-fold higher potency for the former ligand. These findings suggest that Compound **E** binds to P2Y12 at a site that is distinct from that of ticagrelor and that the reaction is dependent on ADP and 2-MeSADP binding.

### 2.5. Selectivity over P2Y1 and P2Y13 Receptors

**Figure 5:**
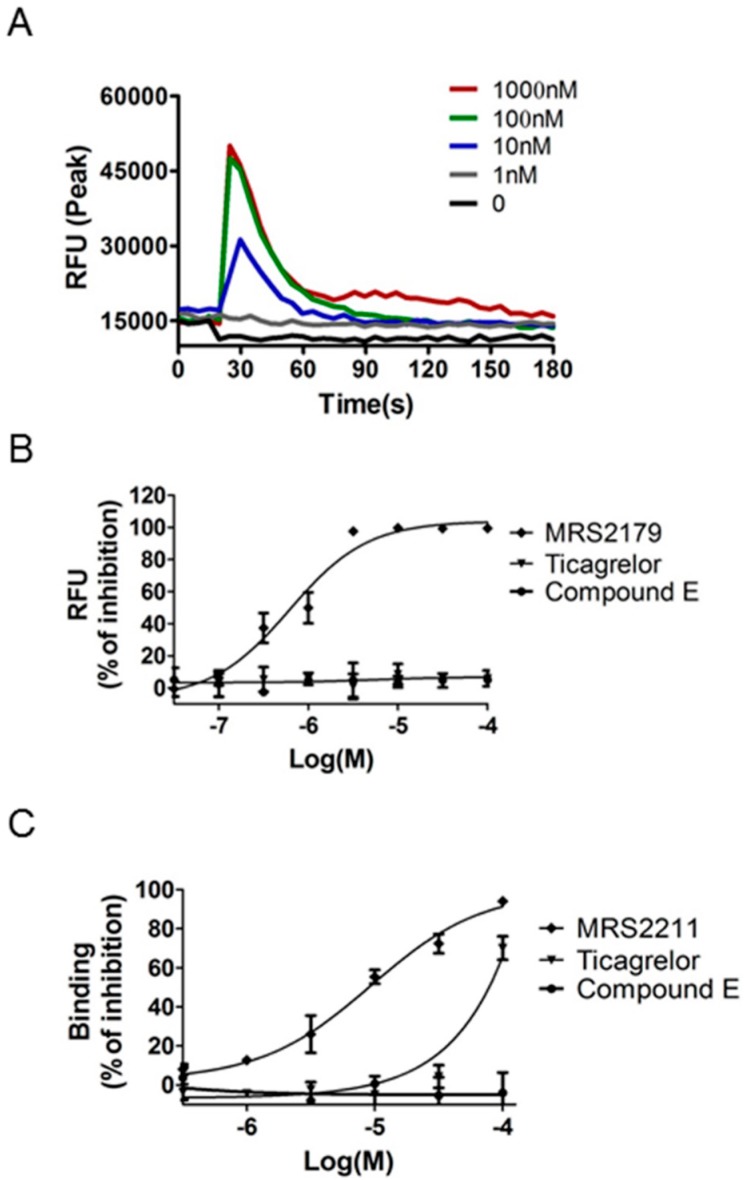
Selectivity for P2Y12 over P2Y1 and P2Y13 receptors (A,B) Changes in cytosolic Ca^2+^ concentration in NIH3T3-rhP2Y1 + Gq cells. (**A**) Representative Ca^2+^ responses triggered by serially diluted MRS2365 in NIH3T3 cells co-transfected with human P2Y1 receptor and Gq/o; (**B**) FLIPR Ca^2+^ dye-loaded NIH3T3-P2Y1 + Gq cells were preincubated with indicated concentrations of MRS2179, 2-MeSAMP, ticagrelor, and Compound **E** (0.03–100 μM). The inhibition of Ca^2+^ increases induced by 100 nM MRS2365 was measured as an increase in relative fluorescence units (RFUs) and is plotted against the log concentration of test compounds; (**C**) Selectivity for rhP2Y13 receptor. Membranes were prepared from COS-7 cells stably expressing the human P2Y13 receptor. Competitive binding was determined using 1 nM [3H]2-MeSADP and Non-specific binding was determined using 25 µM 2-MeSADP. Data are expressed as mean ± SD of one experiment performed in triplicate.

ADP is the cognate agonist of P2Y1, P2Y12, and P2Y13 receptors. To investigate selectivity for P2Y12 over the ADP-activated P2Y1, we evaluated P2Y1-mediated Ca^2+^ mobilization in NIH3T3-P2Y1 + Gq cells by measuring changes in intracellular Ca^2+^ concentration upon treatment with MRS2365, a selective P2Y1 agonist. The EC50 of MRS2365 for P2Y1 receptor activation was 30 nM (Figure 5A). The selective P2Y1 antagonist MRS2179 potently blocked the action of MRS2365 at the P2Y1 receptor (IC_50_ = 0.66 μM), whereas 2-MeSAMP, ticagrelor, and Compound **E** did not exert an inhibitory effect up to 100 μM, indicating that Compound **E** is not a P2Y1 antagonist (Figure 5B). To investigate the selectivity over P2Y13, we evaluated P2Y13-mediated [3H]2-MeSADP binding assay in COS-7-hP2Y13 cells. MRS2211, a selective antagonist for rhP2Y13 receptor, was tested for their ability to inhibit the binding of [3H]2-MeS-ADP to rhP2Y13 (IC_50_ = 9.4 μM). Ticagrelor diplaced the binding of [3H]2-MeS-ADP to rhP2Y13 with >1000-fold selectivity in comparison to rhP2Y12. Compound **E** did not inhibit up to100 μM, indicating that Compound **E** is not a P2Y13 antagonist (Figure 5C).

### 2.6. Docking Study of Compound ***E*** to Human P2Y12 Receptor

**Figure 6:**
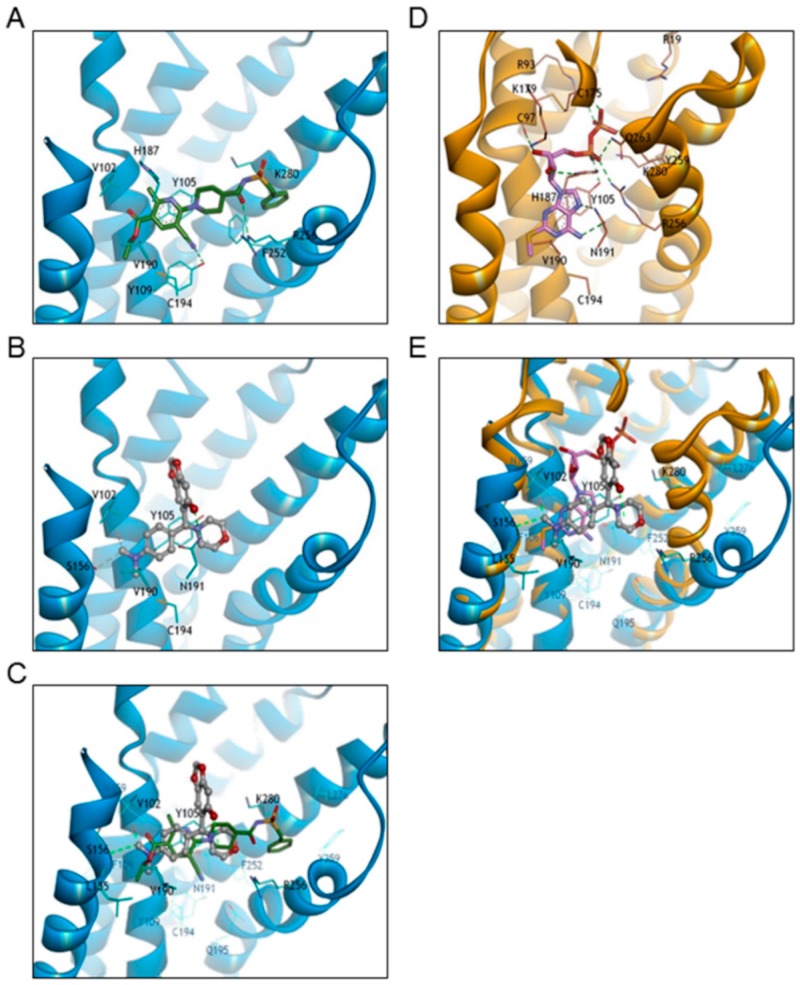
Binding modes of Compound **E**, AZD1283, and 2-MeSADP to hP2Y12. (**A**) Binding mode of AZD1283 (green, stick style) co-crystallized with hP2Y12 (blue ribbon style; PDB code 4NTJ); (**B**) Docking model of Compound **E** (grey, ball-and-stick style) with hP2Y12 (blue ribbon style; PDB code 4NTJ); (**C**) Docking model of Compound **E** overlapping AZD1283 co-crystallized with hP2Y12; (**D**) Binding mode of 2-MeSADP (pink, stick style) co-crystallized with hP2Y12 (orange ribbon style; PDB code 4PXZ); (**E**) Overlay of proposed binding model of Compound **E** with 2-MeSADP co-crystallized with hP2Y12. Hydrogen bonds are displayed as yellow-green dashed lines. For clarity, only key residues are indicated in line-and-stick models and are labeled using the one-letter amino acid code.

Compound **E** is a lead compound identified by high-throughput screening. In order to optimize and improve the pharmacological activity of this compound, we carried out a molecular modeling study in which the binding of Compound **E** was compared to that of reference compounds to evaluate the relationship of their docking modes. To clarify the mode of binding between Compound **E** and human P2Y12 receptor, docking studies were carried out using the X-ray crystal structure of human P2Y12 in complex with its full agonist 2-MeSADP (PDB code 4PXZ) and non-nucleotide antagonist ethyl 6-{4-[(benzylsulfonyl)carbamoyl]piperidin-1-yl}-5-cyano-2-methylpyridine-3-carboxylate (AZD1283; PDB code 4NJT) [14,15]. The *N*,*N*-dimethylaniline moiety of Compound **E** showed the same hydrophobic interactions as the ethyl 5-cyano-2-methylnicotinate of AZD1283 involving Tyr105, Val190, and Leu155 residues. In addition, the methyl group of Compound **E** interacted with weak hydrogen-bonds of the Val102 backbone carbonyl oxygen, and Ser156 side chain containing a hydroxyl group. The morpholine ring of Compound **E** was close to the piperidine of AZD1283; however, the benzodioxole ring had a different binding pocket towards EL2 and the hydroxyl group that formed a hydrogen bond with Asn191 (Figure 6A–C). Compound **E** and 2-MeSADP showed different orientations with only partially overlapping binding pockets. Compound **E** and 2-MeSADP showed overlapping hydrophobic interactions with Tyr105, Leu155, and Val190 residues, the *N*,*N*-dimethylaniline moiety of Compound **E**, and the adenine ring—including the 2-methylthio group—of 2-MeSADP. However, the morpholine ring of Compound **E** had a different binding position with 2-MeSADP, and the benzodioxole ring of Compound **E** was located between ribose and diphosphate groups (Figure 6D,E). These molecular modeling studies suggest that Compound **E** has distinct binding poses with 2-MeSADP and AZD1283, except for common hydrophobic interactions that involve Tyr105, Val190, and Leu155 residues.

## 3. Materials and Methods

### 3.1. Chemical Compounds

4-chloro-1-(3-methoxyphenyl)-6-oxopyridazine-3-carbonitrile (Compound **A**, PubChem CID: 1486955) was purchased from Key Organics Ltd. (Camelford, Cornwall, UK). *N*-cyclopropyl-1-methyl-3-oxo-2,1-benzothiazole-5-sulfonamide (Compound **B**, PubChem CID: 2798315), 5-benzylsulfonyl-4-bromo-2-methylpyridazin-3-one (Compound **C**, PubChem CID: 2774700) and 3-thiophen-2-ylsulfonylpyrazine-2-carbonitrile (Compound F, PubChem CID: 2821421) were purchased from MayBridge, part of Thermo Fisher Scientific Inc. (Tintagel, Cornwall, UK). 1-(4-methoxyphenyl)-3-[(4-methyl-1,2,4-triazol-3-yl)sulfanyl]prop-2-en-1-one (Compound **D**, PubChem CID: 5716610) was purchased from LaboTest OHG. (Niederschoena, Germany). 6-[(4-dimethylamino-phenyl)-morpholin-4-yl-methyl]-benzo[1,3]dioxol-5-ol (Compound **E**, PubChem CID: 339903) was purchased from TimTec LLC. (Newark, NJ, USA). Adenosine 5′-diphosphate (ADP), Adenosine 5′-triphosphate (ATP) and 2-methylthioadenosine 5′-monophosphate (2-MesAMP) were purchased from Sigma-Aldrich Co. (Saint Louis, MO, USA). MRS2179, MRS2211, MRS2365, and 2-MeSADP were purchased from Tocris Bioscience Inc. (San Carlos, CA, USA). Ticagrelor (AZD6140, Brilinta) was provided by LG Life Sciences Ltd. (Daejeon, Chungnam, Korea)

### 3.2. Platelet Preparation for Aggregation Studies

Platelets were isolated from 400 mL whole blood containing 56 mL of CPDA-1 anticoagulant purchased from Chung-Nam Blood Center (Daejeon, South Korea) by centrifugation at 260× *g* for 5 min. The resultant platelet-rich plasma was centrifuged at 370× *g* for 6 min, and the platelet pellet (10^9^/mL) was resuspended in 45 mL plasma and stabilized by agitation for 1 h at 22 °C in blood reservoir. The product was used for experiments within 24 h. Platelet concentrate (PC) was transferred to a new 50-mL Falcon tube and the number of platelets was counted using the ABC Vet blood counter (ABX Diagnostics, Montpellier, France). A 30-mL volume of PCs from the same donor was centrifuged at 1500× *g* for 10 min at room temperature to obtain platelet-poor plasma (PPP). The platelet count was adjusted to 3–4 × 10^8^/mL by diluting with PPP.

### 3.3. Light Transmission Aggregometry

Inhibition of ADP-induced aggregation was assessed in a Costar 96-well flat-bottom plate (Sigma-Aldrich, St. Louis, MO, USA) at room temperature. A 188-µL volume of the diluted platelet was added to each well of the plate along with 2 µL of test compound dissolved in dimethyl sulfoxide (DMSO). The reagents were mixed by placing the plate on a shaker at 1050 rpm for 1 min. A 10-µL volume of 400 μM ADP (final concentration: 20 μM) was added to the wells, and after additional shaking at room temperature for 3 min, the absorption of the samples was read at 595 nm on a Softmax microplate reader (Molecular Devices, Sunnyvale, CA, USA). A dose-response curve was generated using serial dilutions of ADP by adding the vehicle DMSO instead of antagonists in order to calculate the half-maximal effective concentration (EC50) of ADP. Inhibition of aggregation was determined as the increase in absorption at 595 nm after 3 min of incubation relative to absorption values of control preparations (0 and 20 μM ADP without antagonists). Sigmoidal dose-response curves and half-maximal inhibitory concentration (IC_50_) were derived by non-linear regression analysis using Prism 5.01 software (GraphPad, San Diego, CA, USA).

### 3.4. Cell Culture

The HEK293T, NIH3T3, and COS-7 cell lines were purchased from American Type Culture Collection (Manassas, VA, USA). The human cDNA clones of P2Y1 (NM_002563) and P2Y12 (NM_022788) were purchased from UMR cDNA resource center (Rolla, MO, USA). The human cDNA clone of P2Y13 (NM_023914.2) was purchased from OriGene Technologies, Inc. (Rockville, MD, USA). The pDisplay vector and Gq alpha plasmid DNA was obtained from Dr. Hee Dong Park. HEK293T cells stably expressing rhP2Y12 were grown in Dulbecco′s Modified Eagle′s Medium (DMEM) containing 10% fetal bovine serum (FBS), 1% antibiotics, and 0.5 mg/mL geneticin. NIH3T3 cells stably expressing rhP2Y1 + Gq receptor were grown in DMEM containing 10% FBS, 1% antibiotics, 0.5 mg/mL geneticin, and 0.4 mg/mL zeocin. COS-7 cells stably expressing rhP2Y13 were grown in DMEM containing 10% fetal bovine serum (FBS), 1% antibiotics, and 0.5 mg/mL geneticin.

### 3.5. Human P2Y12 Receptor and P2Y13 Receptor-Binding Assay with [3H]2-Methylthioadp and [3H]ADP

The pDisplay-human P2Y12 DNA clone and pDisplay-human P2Y13 DNA clone were stably expressed in HEK293T and COS-7 cells, respectively; recombinant human (rh)P2Y12- and rhP2Y13-expressing cells were resuspended in hypotonic buffer consisting of 10 mM HEPES, 10 mM NaCl, 1 mM EDTA, 1 mM EGTA (pH 7.4), and protease inhibitor cocktail. Following 15-min incubation on ice, cells were homogenized on ice using a glass/teflon homogenizer (1000 rpm, 10 strokes). The homogenate was centrifuged at 500 × *g* for 15 min and the supernatant was centrifuged at 100,000× *g* for 30 min. The membrane pellet was adjusted to 1 mg/mL with membrane buffer consisting of 10 mM HEPES, 5 mM KCl, and 130 mM NaCl (pH 7.4) and stored at −80 °C. A 100-μL volume of 0.2 mg/mL membrane protein was added to each well of a 96-well plate along with 25 μL of test compound and [3H]2-MeSADP or [3H]ADP for a total reaction volume of 0.15 mL/well. The plate was incubated for 1 h at room temperature. The reaction was terminated by passing the solution through a GF/B filter presoaked with 0.1% polyethyleneimine using a 96-well cell harvester. The filter was washed 10 times with ice-cold wash buffer consisting of 10 mM HEPES (pH 7.4) and 138 mM NaCl. Radioactivity of the P2Y12 receptor or P2Y13 receptor bound to the membrane were measured using a scintillation TopCount instrument (PerkinElmer, Boston, MA, USA). Total binding was determined from the counts per minute (CPM) of the test group with no compound and non-specific binding was determined from the CPM of the test group containing 2-MeSADP (25 μM) and ADP (100 μM). Percent inhibition of binding was calculated as follows: (100—(specific binding in the presence of test material/specific binding in the absence of test material × 100)).

### 3.6. Ca^2+^ Mobilization Assay

Intracellular Ca^2+^ mobilization was analyzed using the FLIPR 5 assay kit as previously described [16]. Briefly, pDisplay-human P2Y1 DNA clone and pcDNA-Gq DNA clone were stably expressed in NIH3T3 cells. NIH3T3-P2Y1 + Gq cells (3 × 10^5^ cells/mL) were seeded in a 96-well plate and allowed to adhere overnight. An equal volume of FLIPR 5 assay kit loading buffer (Molecular Devices) was added directly to the culture medium for 1 h at room temperature, followed by 20-min incubation after adding the test compounds. Changes in intracellular Ca^2+^ were measured with a FlexStation II scanning fluorometer (Molecular Devices).

### 3.7. Molecular Modeling

To clarify the binding mode of compounds to the human P2Y12 receptor, we carried out a flexible docking analysis using the Schrödinger Glide v.6.6 program with standard precision settings (Schrödinger LLC, New York, NY, USA). The two X-ray co-crystal structures of hP2Y12 were obtained from the Protein Data Bank (PDB; codes 4NTJ and 4PXZ, respectively) (http://www.rcsb.org). Ligands were minimized using a Merck Molecular Force Field (MMFF) with a dielectric constant of 80.0 using the MacroModel v.10.7 program. We selected a binding model based on the best docking score and visual inspection. All molecular graphics figures were generated using the visualizer in Discovery Studio v.4.5 (Biovia, San Diego, CA, USA).

### 3.8. Statistical Analysis

Data are shown as means ± SD and were analyzed using GraphPad Prism v.5.01 software (GraphPad Inc., La Jolla, CA, USA). *p* < 0.05 was considered statistically significant.

## 4. Discussion

ADP is a platelet agonist that plays an important role in hemostasis and pathophysiological arterial thrombosis. ADP activates platelets through three purinergic receptors—namely P2Y1, P2Y12, and P2X1—causing platelets to undergo changes in shape, as well aggregate and generate thromboxane A2. Of 6211 commercially available compounds, 43 inhibited ADP-induced platelet aggregation. However, only Compound **E** selectively antagonized P2Y12 at micro-molar concentrations. The platelet aggregation experiments were based on a 96-well plate method, and the data reflects only the late phase of aggregation; indeed, P2Y1 remained functional in the presence of Compound **E**, which did not alter platelet shape. Thus, Compound **E** is a direct-acting, reversible P2Y12 inhibitor like ticagrelor but with pharmacologically distinct characteristics. Firstly, ticagrelor inhibited ADP-induced platelet aggregation in a time-dependent manner, but the pre-incubation step did not increase the potency of Compound **E** (data not shown). Secondly, Compound **E** has partially overlapping binding pocket with 2-MeSADP, whereas ticagrelor binds to P2Y12 at an allosteric site that is separate from the 2-MeSADP-binding site. However, it remains to be determined whether Compound **E** offers any therapeutic benefits over ticagrelor. The absolute configuration at the chiral center of Compound **E** has not been defined in here; we, therefore, calculated the docking scores of both *R* (−6.44) and *S* (−6.19) configurations. We found a slightly higher docking score for the *R* than for the *S* configuration and, therefore, the docking model for Compound **E** is presented as the *R* configuration. The docking study showed that compound **E** could bind to the active site of the P2Y12 receptor, suggesting in vitro activity. A comparison of the proposed binding model of compound **E** (grey, ball and stick style) with that of AZD1283 (green, stick style) and 2-MeSADP (pink, stick style) revealed hydrophobic interactions of compound **E** when they were superimposed on reference compounds (left side in Figure 6), although the hydrophilic interaction site of compound **E** had a binding mode that differed from that of references (right side in Figure 6). These results indicate that the distinct binding mode of compound **E** is responsible for its weaker in vitro activity relative to 2-MeSADP and AZD1283. Nonetheless, the activity of compound **E**—which has a new scaffold—can be optimized by structure-based drug design.

In conclusion, our results demonstrate that Compound **E** is a novel morpholine moiety scaffold compound that is direct-acting and pharmacologically distinct from ticagrelor, and reversibly antagonizes P2Y12. These findings provide an opportunity for the development of a new class of P2Y12 antagonist exhibiting anti-platelet function.

## Figures and Tables

**Table 1:** Inhibition of ADP-induced platelet aggregation of selected compounds.

Ligand	20 μM ADP (IC_50_, μM)	Compounds	20 μM ADP (IC_50_, μM)
ATP	2.14 ± 0.51	**A**	8.04 ± 0.51
MRS2179	1.1 ± 0.01	**B**	9.49 ± 1.15
2-MeSAMP	1.09± 0.16	**C**	4.03 ± 0.95
Ticagrelor	0.32 ± 0.02	**D**	6.08 ± 0.11
		**E**	6.21 ± 0.71
		**F**	9.35 ± 2.19

**Table 2:** Competitive binding affinities of selected compounds for rhP2Y12 receptor.

Compounds	IC_50_ vs. [^3^H]-2-MeSADP (μM)	IC_50_ vs. [^3^H]ADP (μM)
**A**	>30	>30
**B**	>30	>30
**C**	>30	>30
**D**	>30	>30
**E**	15.51 ± 2.24	8.33 ± 1.69
**F**	>30	>30

**Table 3:** Comparison of binding affinities against [^3^H]-2-MesADP and [^3^H]ADP ligands.

Ligand	IC_50_ vs. [^3^H]-2-MeSADP (μM)	IC_50_ vs. [^3^H]ADP (μM)
2-MeSADP	0.12 ± 0.02	17.22 ± 4.62
ADP	24.78 ± 2.09	0.19 ± 0.10
Ticagrelor	0.04 ± 0.02	8.40 ± 3.05
Compound **E**	15.51 ± 2.24	8.33 ± 1.69
